# Request for public discussion and ballot to amend SeqCode rules on priority of *Candidatus* names and correction of typographic and orthographic errors

**DOI:** 10.1038/s43705-023-00303-y

**Published:** 2023-09-14

**Authors:** William B. Whitman, Brian P. Hedlund, Marike Palmer, Iain Sutcliffe, Maria Chuvochina

**Affiliations:** 1 Department of Microbiology, University of Georgia, Athens, GA USA; 2 School of Life Sciences and Nevada Institute of Personalized Medicine, University of Nevada, Las Vegas, Las Vegas, NV USA; 3 School of Life Sciences, University of Nevada, Las Vegas, Las Vegas, NV USA; 4 Faculty of Health and Life Sciences, Northumbria University, Newcastle upon Tyne, UK; 5 The University of Queensland, School of Chemistry and Molecular Biosciences, Australian Centre for Ecogenomics, St Lucia, QLD Australia

**Keywords:** Microbiome, Biodiversity

## Introduction

The Code of Nomenclature of Prokaryotes Described from Sequence Data, or SeqCode, is a new code of nomenclature in which genome sequences are the nomenclatural types of species [1, 2]. While similar to the International Code of Nomenclature of Prokaryotes (ICNP, [3]) in many aspects, the SeqCode does not require deposition of type strains into international culture collections. Thus, it allows formation of stable and precise names for uncultured and fastidious prokaryotes that cannot practically be deposited, preserved, or distributed as viable pure cultures. Because the diversity of uncultured prokaryotes greatly exceeds that of readily culturable prokaryotes, the SeqCode is necessary and appropriate for naming the majority of prokaryotic species, enabling effective communication and unification of taxonomies of uncultured and cultured prokaryotes. The start date of the SeqCode was January 1, 2022, and the online SeqCode Registry (https://seqco.de) was created to manage the validation process and serve as the official database of validly published names [4]. During the initial year of implementation of the SeqCode, problems were encountered with the validation of some published *Candidatus* names. In addition, there was a lack of clarity on the authority of the SeqCode Registry curators to correct names with typographic and orthographic errors. Amendments are proposed to address these issues.

Amendments to the SeqCode must be proposed by publication of a peer-reviewed article, as outlined in Article 11 of the SeqCode Statutes [5]. According to these Statutes, the rationale for the proposed changes and new wording must be clearly stated. Following publication, the Chair of the SeqCode Legislative Commission will initiate a public discussion of the proposal for a period of not less than 3 months and not longer than 6 months. Following the authors’ opportunity to respond, their response and the public discussion will be compiled by the Secretary of the Legislative Commission and communicated to the Secretary of the Executive Board within 1 month. The Executive Board will then disseminate the materials prepared by the Legislative Commission and arrange for a ballot of the SeqCode Community.

## Proposed amendments to address problems related to conflicts between published *Candidatus* names and the SeqCode rules of priority

In its effort to create a stable nomenclature based on genome sequences as nomenclatural types of prokaryotic species, the SeqCode steering group failed to make provisions for the legacy of *Candidatus* names. *Candidatus* status is used for names of uncultured taxa as outlined in Appendix 11 of ICNP [3]. Over a thousand such names have accumulated since *Candidatus* status was proposed in the mid-1990s [6, 7]. However, the recommendations on the use of *Candidatus* names are vague, and these provisional names are not eligible for validation until they satisfy the requirements of the ICNP, the primary one being deposition of cultures [3, 8]. Even though *Candidatus* names do not have an official status in nomenclature, many are widely used in the literature, and it is desirable to preserve such names and validate them under the SeqCode when suitable nomenclatural types are available.

Following from Rules 16, 22, 23b and 23d of the SeqCode, names of taxa above the rank of genus must be formed from the stem of the earliest legitimate genus name, where the corresponding genus is treated as the type genus [1]. As a consequence of these rules, existing *Candidatus* phylum, class, order, and family names are “unprotected” until the corresponding type genus name is validly published. In other words, if another genus name belonging to these higher taxa were to be validated before validation of the *Candidatus* type genus name, it would lead to formation of different names for these higher taxa and render the established *Candidatus* higher taxa names ineligible for validation. One solution to this problem would be to immediately validate the type genus names for the existing *Candidatus* higher taxa. However, this solution is impractical for a number of reasons: (i) it is a time-consuming task for which resources have not been allocated; (ii) many *Candidatus* names of higher taxa, particularly phyla, were published without designating a type genus, so one must be designated; (iii) many *Candidatus* names of higher taxa contain subordinate taxa, but there is no genus with a stem that corresponds with the names of the higher taxa; (iv) genomic data that could serve as nomenclatural types are not publicly available or are below the recommended genome quality standards outlined in Appendix 1 of the SeqCode [1, 9].

To provide an opportunity to retain the *Candidatus* names of higher taxa, we propose to amend the SeqCode to allow exceptions to the rules of priority for ranks above the genus in cases where an effectively published *Candidatus* name exists. In recognition of the possibility that some *Candidatus* names may be of little value or based on flawed evidence, we propose that these exceptions expire on January 1, 2027. This date was chosen to allow sufficient time for investigators to register *Candidatus* names of value to the research community. Because many of these names have already been effectively published and type genomes of sufficient quality are available, they may be simply entered into the SeqCode Registry. In some cases, it will be necessary to publish suitable genus names for deriving the higher taxa names, and time is allowed for these publications. If a type genome is not currently available, sufficient time is allowed to either perform the necessary experiments to obtain a type genome of high quality or request an exemption from the SeqCode Committee. After that date, the normal rules of priority will be followed, and *Candidatus* names that have not been validated will compete with other names for priority upon their validation. This amendment will be retroactive to the start date of the SeqCode, January 1, 2022.

Under this amendment, it will be possible to name subordinate taxa belonging to higher taxa and retain *Candidatus* names until they can be validated. Different actions would then be needed before January 1, 2027 to preserve *Candidatus* names depending on the specific problems described above (i–iv, above). It is anticipated that these actions will be initiated by the authors, SeqCode Committee members, or the broader community.

To achieve this goal, Rule 23d of the SeqCode needs to be amended. The proposed amendments are given below. Arguments for and against the amendment of Rule 23d are provided in Box 1.

Box 1 Arguments for and against emendation of Rule 23d **Arguments for**: As currently implemented, validation of a genus name belonging to a taxon of a rank higher than genus with an existing *Candidatus* name would automatically replace the *Candidatus* name with the name derived from the stem of the validated genus name. In these cases, all *Candidatus* names that have not been validated under the SeqCode will not obtain standing in nomenclature. The amendment described here would provide time for the SeqCode Committee and research community to validate many *Candidatus* names, with modifications where necessary, to ensure their permanence under the SeqCode. The research community benefits from stability offered by validation of names already established in literature.

**Arguments against**: As currently implemented, the SeqCode offers a clean start and would ignore the backlog of *Candidatus* names that have not been or cannot yet be validated under the SeqCode or ICNP. In this scenario, authors can generate new names for all taxa that would be free of the complications of existing *Candidatus* names, many of which are problematic and require modification, such as identification of appropriate nomenclatural types and/or submission of raw and assembled data to INSDC databases. This would also provide the best opportunity to designate high-quality nomenclatural types for all taxa, independent of their legacy in literature, implement community-driven quality standards, and limit the number of exceptions that might be requested to validate existing *Candidatus* names.

## Rule 23d [original rule]

The priority date of names of taxa of rank higher than genus proposed after 1 January 2022 is the same as the priority date of the corresponding type genus name. The priority date for names published before 1 January 2022 is the same as their priority under the ICNP.

## Rule 23d [amended rule, new text underlined]

The priority date of names of taxa of rank higher than genus proposed after 1 January 2022 is the same as the priority date of the corresponding type genus name unless there is a published *Candidatus* name for the taxon. In that case, the date of publication of the *Candidatus* name establishes priority for the *Candidatus* name upon validation unless there is an ICNP name validly published for the same taxon before 1 January 2022, in which case the date of valid publication of the ICNP name establishes priority. Validating existing *Candidatus* higher taxa names requires validation of a type genus name with an appropriate stem and the associated names for the subordinated higher taxonomic ranks where appropriate. However, this protection of *Candidatus* names is lost if the *Candidatus* name is not validated by 1 January, 2027. The priority date for names published before 1 January 2022 is the same as their priority under the ICNP. This rule is retroactive to 1 January, 2022.

Note 1. After 1 January 2027, it is strongly recommended that genus names are proposed to preserve published *Candidatus* names for higher taxa, when appropriate, to allow validation of existing *Candidatus* names.

## Proposed amendments to clarify procedures for correction of typographic and/or orthographic errors during registration of SeqCode names

Another common problem in prokaryotic nomenclature is that errors can occur during the formation of names that are not corrected during peer review of the effective publication. Currently, both the ICNP and the SeqCode require that names are formed in accordance with the rules of Latin grammar. Under the ICNP, names containing typographic and orthographic errors are often validated and subsequently corrected either by the original authors or other authors [3], and SeqCode follows similar rules (Rule 48). Under Rule 48, errors affecting the stem of a type genus name or the gender of a specific epithet must be corrected. These types of errors are common and require corrections as they affect formation of higher taxa names or specific epithets when a species is transferred to a different genus. The following amendments are proposed to clarify the responsibility of the SeqCode Registry curators to correct typographic and orthographic errors prior to validation of a name. This capability is similar to that of the List Editors of the International Journal of Systematic and Evolutionary Microbiology to correct imperfectly formed names in the Validation Lists that summarize names formed under the ICNP [10].

As currently written, Rule 48 describes procedures for correcting typographic and orthographic errors for names that have already been validated, but it does not clearly describe how those errors should be handled in names that are submitted for validation. This lack of clarity could lead the SeqCode Registry curators to not validate names containing typographic and orthographic errors, which limits progress and could lead to frustration among the research community. These amendments provide the necessary clarity and give the SeqCode Registry curators authority to validate names and make necessary corrections during the validation process. This process would allow documentation of implemented corrections in the SeqCode Registry and would provide a clear record from the spelling in the initial effective publication to the corrected names. By promptly correcting errors during or after registration, the persistence of incorrect names in the literature will be limited. Arguments for and against the amendment of Rule 48 are provided in Box 2.

Box 2 Arguments for or against emendation of Rule 48 **Arguments for**: As currently written, Rule 48 describes procedures for correcting typographic and orthographic errors for names that have already been validated, but it does not clearly describe how SeqCode Registry curators should deal with names with typographic and orthographic errors that are submitted for validation. These amendments provide the needed clarity and give the SeqCode Registry curators authority to validate names with errors and make necessary corrections during or after the validation process. This process would allow documentation of implemented corrections in the SeqCode Registry and would provide a clear record for users from the spelling in the initial effective publication to the corrected names. By promptly correcting errors during or after registration, the persistence of incorrect names in the literature will be limited.

**Arguments against**: Although the SeqCode requires that names conform to proper Latin and use standard suffixes, some authors may disagree with the corrections proposed by the SeqCode Registry curators.

## Rule 48 [original]

The original spelling of a name or epithet must be retained, except for typographic or orthographic errors.

An unintentional typographical or orthographic error later corrected by the author is to be accepted in its corrected form without affecting the status and date of valid publication. It can also be corrected by a subsequent author who may or may not mention that the spelling is corrected. However, the abbreviation “corrig.” (*corrigendum*) may be appended to the name if an author wishes to draw attention to the correction. Succeeding authors may be unaware that the original usage was incorrect and use the spelling of the original author(s). Other succeeding authors may follow the correction of a previous author or may independently correct the spelling themselves, but in no case is the use of corrig. regarded as obligatory. None of these corrections affects the status and date of validation.

Note. The liberty of correcting a name or epithet must be used with reserve, especially if the change affects the first syllable and above all the first letter of the name or epithet.

## Rule 48 [amended rule, new text underlined]

The original spelling of a name or epithet must be retained, except for typographic or orthographic errors.

An unintentional typographical or orthographic error in an effectively published name may be corrected before or after validation in the Registry by the author or the SeqCode Registry curators. The name is to be accepted in its corrected form without affecting the status and date of valid publication. It can also be corrected by a subsequent author in a peer-reviewed publication. That publication may or may not mention that the spelling is corrected. In that case, the authors or SeqCode Registry curators must update the name in the SeqCode Registry. [deleted:However,] The abbreviation “corrig.” (*corrigendum*) may be appended to the name if an author wishes to draw attention to the correction. Succeeding authors may be unaware that the original usage was incorrect and use the spelling of the original author(s). Other succeeding authors may follow the correction of a previous author or may independently correct the spelling themselves, but in no case is the use of corrig. regarded as obligatory. None of these corrections affects the status and date of validation.

Note 1. The liberty of correcting a name or epithet must be used with reserve, especially if the change affects the first syllable and above all the first letter of the name or epithet.

Note 2. When feasible, as a courtesy, the SeqCode Registry curators will inform one or more authors of the effective publication that typographical or orthographic errors have been corrected in the Registry.

## In summary

We welcome public discussion of these proposed amendments to the SeqCode followed by a balloting of the SeqCode Community. Those interested in these issues are invited to join the SeqCode Community via the SeqCode Community Signup form (https://seqco.de/join).
